# Artificial Intelligence and Its Application to Minimal Hepatic Encephalopathy Diagnosis

**DOI:** 10.3390/jpm11111090

**Published:** 2021-10-26

**Authors:** Jakub Gazda, Peter Drotar, Sylvia Drazilova, Juraj Gazda, Matej Gazda, Martin Janicko, Peter Jarcuska

**Affiliations:** 1 2nd Department of Internal Medicine, PJ Safarik University and Louis Pasteur University Hospital, 040 11 Kosice, Slovakia; jakub.gazda@upjs.sk (J.G.); drazilovasylvia@gmail.com (S.D.); peter.jarcuska@upjs.sk (P.J.); 2 Intelligent Information Systems Lab, Faculty of Electrical Engineering and Informatics, Technical University of Kosice, 040 01 Kosice, Slovakia; peter.drotar@tuke.sk (P.D.); juraj.gazda@tuke.sk (J.G.); matej.gazda@tuke.sk (M.G.)

**Keywords:** artificial intelligence, psychometric tests, minimal hepatic encephalopathy, covert hepatic encephalopathy

## Abstract

Hepatic encephalopathy (HE) is a brain dysfunction caused by liver insufficiency and/or portosystemic shunting. HE manifests as a spectrum of neurological or psychiatric abnormalities. Diagnosis of overt HE (OHE) is based on the typical clinical manifestation, but covert HE (CHE) has only very subtle clinical signs and minimal HE (MHE) is detected only by specialized time-consuming psychometric tests, for which there is still no universally accepted gold standard. Significant progress has been made in artificial intelligence and its application to medicine. In this review, we introduce how artificial intelligence has been used to diagnose minimal hepatic encephalopathy thus far, and we discuss its further potential in analyzing speech and handwriting data, which are probably the most accessible data for evaluating the cognitive state of the patient.

## 1. Introduction

The European Association for the Study of the Liver (EASL) and the American Association for the Study of Liver Diseases (AASLD) defined hepatic encephalopathy (HE) as a brain dysfunction caused by liver insufficiency and/or portosystemic shunting; it manifests as a spectrum of neurological or psychiatric abnormalities ranging from subclinical alterations to coma [1]. Hepatic encephalopathy should be classified according to the severity of clinical manifestation as either covert (CHE) or overt (OHE) (ISHEN classification; International Society for Hepatic Encephalopathies and Nitrogen Metabolism) or as Minimal HE (MHE) and grade I–IV HE (WHC; West Haven criteria), while the term “covert” includes both minimal and grade I HE [2,3,4]. MHE affects the lives of both patients and caregivers by lowering the quality of life and worsening socioeconomic status. Cognitive decline, attention deficit, loss of working memory, and visuomotor coordination also lead to higher frequency of falls and driving accidents. This review focuses on the diagnostic approach to the minimal hepatic encephalopathy, which is a challenging subject with large areas of uncertainty on the one hand and pronounced clinical needs and consequences on the other. We discuss several studies which employed means of artificial intelligence in diagnosing MHE, and we propose an artificial intelligence analysis of common behavioral patterns, such as speech or handwriting, as a possible screening option for MHE. The current article also provides the point of view of several specialists in cognitive computation and decision support systems (authors PD, JuG, MG).

## 2. Pathogenesis

The pathogenesis of HE is complex and not yet fully elucidated. In patients with liver failure, hyperammonemia is present and is considered to play a significant role in the pathogenesis of HE. Ammonia can cross the blood-brain-barrier in the form of uncharged ammonia or the ionized NH4+ form by passive diffusion and active transport, respectively. After crossing the blood-brain-barrier, ammonia preferentially enters astrocytes and induces alteration of the pH, membrane potentials and electrolyte balance; inhibits the tricarboxylic acid cycle; impairs mitochondrial permeability and thus leads to cell dysfunction [5]. After entering astrocytes, the ammonia is detoxicated through the generation of glutamine, which acts as an osmole; it therefore enhances water diffusion, which ultimately leads to brain oedema. HE is further deteriorated by the presence of hyponatremia and microglial cell activation [6,7]. Patients with hyperammonemia and no systemic inflammation suffered significantly less from HE when compared to those with concomitant systemic inflammation. TNF-α is considered responsible for this [6,8]. The gut-liver axis plays a significant role in systemic inflammation in patients with liver cirrhosis, although other infection foci are frequently present as well [9,10].

Furthermore, alteration in neurotransmission contributes to the pathogenesis of HE as well. Decreased glutaminergic transmission and increased GABA-ergic transmission happens via multiple steps, and both still remain to be fully elucidated. Neuronal disinhibition in the monoamines system has also been associated with HE [8]. Finally, manganese deposits have been described as another cofactor in the development of HE [8].

## 3. Clinical Picture

HE represents a wide spectrum of neurological or psychiatric abnormalities ranging from subclinical alterations to coma [1]. Patients with HE present with an attention deficit as well as lethargy and apathy. Furthermore, motoric functions are impaired, and patients can present with asterixis (flapping tremor), apraxia or dysgraphia. The evaluation of such changes is important in diagnosing and classifying hepatic encephalopathy. There are several different classifications of HE according to the severity of clinical manifestation [2,3,4,11,12,13]. The West Haven criteria are the most commonly used in clinical practice; however, they are subject to both inter- and intra-rater variability (Table 1) [3]. The less subjective ISHEN classifies HE according to its clinical presentation as either covert or overt (Table 1) [2].

OHE is a diagnosis based on a clinical manifestation (WH/ISHEN) when other potential causes of neuropsychiatric impairment have been ruled out. On the other hand, there are either no or only very subtle clinical signs of CHE or MHE, respectively. Furthermore, in patients with OHE, a probable trigger temporally associated with an OHE episode can usually be identified. In contrast, covert hepatic encephalopathy is usually more dependent on liver dysfunction and/or portosystemic shunting than on an obvious trigger [14]. Differences between minimal and grade I HE are even more subtle. The main distinction is that, in patients with grade I HE, some small cognitive or behavioral impairment may be found even without specialized testing, particularly by caregivers or family members, who know the patient well and can observe his/her behaviour longitudinally, which is not the case in MHE, where the diagnosis can only be made on the basis of specialized tests.

MHE prognosticates both OHE development and liver-related mortality. Furthermore, it is associated with decreased health-related quality of life and with increased risk of car accidents [15,16]. Thus, patients with liver cirrhosis, particularly active drivers, should be screened for MHE regularly, and all these tests should be performed by a trained examiner adhering to recommendations [16]. However, such a strategy would be relatively expensive [1].

## 4. Diagnosis of Minimal Hepatic Encephalopathy

The diagnosis of this condition should be performed as follows. First, the physician should quantify the neuro-psychiatric impairment using any of the validated tests (Section 4.1). If patient is diagnosed with neuro-psychiatric impairment, the physician should rule out alternative causes of brain dysfunction (causes related to diabetes or alcohol intake, medication side effects and other less common causes), because HE of any grade still remains a diagnosis of exclusion [1]. Therefore, adequate measures including a thorough history, laboratory and imaging tests and psychiatric evaluation are warranted as clinically indicated. Finally, the observation of specific changes in the CNS physiology can confirm the diagnosis of MHE; however, it is not a strict requirement (Section 4.2).

Neuro-psychiatric impairment is a finding sine qua non for MHE, however its discovery and measurement in clinical practise are often challenging. Many psychometric and neurophysiological tests have been developed to address this challenge, however there are several problems. First, these tests need to be nationally and culturally validated. Second, these tests measure only certain aspects of a global neuro-psychiatric function and may miss abnormalities in other aspects. Third, these tests are time-consuming, and therefore not completely feasible in clinical practice. Currently, there is no gold standard for the diagnosis of this syndrome [17] and rather few specialized centers indeed screen for the presence of MHE in out-patients’ cirrhotics. Furthermore, the International Society for Hepatic Encephalopathy and Nitrogen Metabolism advises against the use of a combination of two or more tests to establish the diagnosis, because of the lack of clinical rationale or available evidence to substantiate it [16]. In this part, we introduce the most frequently used tests for diagnosing minimal hepatic encephalopathy. Together with the mechanisms of these tests, we also report their sensitivity and specificity values. However, we need to highlight that direct comparison of these metrics is impossible because these studies’ reported diagnostic performance was compared to different standards.

### 4.1. Paper-Pencil and Computerized Psychometric Tests

#### 4.1.1. Psychometric Hepatic Encephalopathy Score (PHES)

The psychometric hepatic encephalopathy score (PHES) is a battery of five bedside paper-pencil tests (number connection test A (NCT A), number connection test B (NCT B), the line tracing test, the serial dotting test, and the digit symbol test) [18]. In the NCT, patients are asked to connect numbers with one another in the correct sequence. In the line tracing test, patients are asked to draw a line in between two lines running parallel with one another and to do it without crossing either one of these two lines. In serial dotting tests, patients are asked to place a dot inside each circle (ten rows consisting of ten circles). Finally, in digit symbol tests, patients are asked to write down the corresponding symbol under each digit, while digit-symbol pairs are presented on each test paper. PHES examines motor speed and accuracy, visual perception, visuospatial orientation, visual construction, attention, and, to a lesser extent, memory, and it takes 10–20 min to carry out [18]. PHES tests should be standardized in a healthy control population prior to their use in clinical practice, and the mean score of each and all tests should be calculated together with respective standard deviations (SD). Individual test results in between the −1 SD and +1 SD interval should be scored with 0 points; those between −1 SD and −2 SD should be scored with −1 point; those between −2 and −3 SD should be scored with −2 points; and those worse than −3 SD should be scored with −3 points. Results better than the +1 SD should all be scored with only 1 point. Finally, since the line tracing test has two different outcomes, patients can achieve between −18 to +6 points. Patients with liver cirrhosis and with no clinical manifestation of HE are diagnosed with MHE if they achieve a total score of less than −4 [18]. PHES has been adapted in many different countries (China, India, Italy, Iran, Korea, Mexico, Poland, Portugal, Romania, Spain, and Turkey) [19,20,21,22,23,24,25,26,27,28,29]. The PHES battery of 5 tests is currently considered to be the best clinical standard for the diagnosis of MHE [25]. In general, PHES is considered to be a time-consuming test with suboptimal sensitivity, thus it is not used in clinical practice frequently.

The NCT-A alone is arguably the most frequently used test to diagnose MHE. It takes 60 s (±36 s) to complete. However, few data are available about its performance as a standalone test compared to either full PHES battery or EEG in MHE. In one study from China, NCT yielded a sensitivity of 87% and specificity of 94%, when compared to the pathological result of a general purpose IQ test [30].

#### 4.1.2. EncephalApp_Stroop

The Stroop test is a novel point-of-care assessment tool of psychomotor speed and cognitive flexibility. The Stroop test is based on correctly identifying the colour that a stimulus is presented in. Its assessment relies on two kinds of stimuli (number signs—# and three nouns representing different colours–red, green, and blue). Both stimuli are presented in one of three different colours (again red, green, and blue). The goal is to correctly identify the colour that the stimulus is presented in. However, there are two states to the test. The “on” and the “off” state. The “off” state is considered to be easier. However, during the more challenging “on” state, there is actually discordance between the word representing a particular colour and the actual colour that the word is displayed in. For instance, the word “red” is displayed in the blue colour, and thus the patient must mark the blue option, not the red option, and do so as quickly as possible. If the patient makes a mistake, the run stops and the patient must start again. EncephalApp_Stroop is an application that provides this test, it can be freely downloaded from both the App Store and Google Play, however, although being completely electronic, it does not employ any means of artificial intelligence. The outcomes are total time and number of runs needed to complete five correct runs in both the “on” and “off” states. EncephalApp_Stroop has been translated to Arabic, Danish, French, German, Hungarian, Indonesian, Italian, Mandarin, Portuguese, Romanian, Slovakian, Spanish, Tami, and the Thai language. Screening for MHE using applications like EncephalApp_Stroop does not require trained medical staff to interpret the results and reduces the time required to complete such screening [31].

The EncephalApp_Stroop has been validated for screening for MHE [32,33]. Interestingly, in all patients with liver cirrhosis, total time in the “off” state had highest AUROC (91.0%, the sensitivity and specificity were 94% and 79%, respectively) when compared to standard psychometric tests [32]. Similarly, total time in the “off” state had highest AUROC (0.87) in patients with liver cirrhosis but without a previous bout of OHE [32]. Bajaj et al. confirmed its external validity, face validity, and test-retest reliability [34]. EncephalApp_Stroop has been validated in Brazil, China and Korea, and was further expanded in the USA [31,33,35,36].

#### 4.1.3. Other Strategies Adapted for Testing for MHE

The animal naming test (ANT) is another test implemented for diagnosing MHE. Here, a patient is asked to list a maximum number of animals in one minute. Campagna et al. adjusted this test to both age and education of patients and found that a cut-off of 15 animals discriminated between unimpaired cirrhotic patients and patients with MHE/HE I [37]. There are also computerized tests adapted for testing for MHE. For instance, critical flicker frequency (CFF) is a physiological tool which assess the threshold frequencies at which light pulses are perceived as fused or flickering light. It is not influenced by either the age or the education level of a patient. CFF assesses visual function and general arousal [38]. In the inhibitory control test (ICT) the patient is instructed to respond only when the X and Y on display are alternating and to inhibit from responding when X and Y are not alternating. The test analyses patients’ reaction speed, attention, and inhibitory control. During a continuous reaction time (CRT) test, a series of auditory stimuli are delivered to a patient at random intervals. Each time the patient hears a sound, he or she is supposed to press a button. The test analyses the patient’s reaction speed, attention, and inhibitory control [39]. Table 2 summarizes the diagnostic performance and the time required to perform the above mentioned tests. 

### 4.2. Detection of Changes in Brain Physiology

Changes in brain physiology are even more difficult to detect than neuro-psychiatric impairment in patients with MHE. Electroencephalograms (EEG) have been used for a long time to diagnose MHE. Currently however, classical subjective analysis of EEG is considered to be only a complementary test in cirrhotic patient with neuro-psychiatric impairment since it may shift the diagnosis towards other aetiologies [40]. Because of this and the logistics issues (the need for evaluation of EEG by a neurologist), the EEG is not routinely employed for diagnosing minimal hepatic encephalopathy in clinical practice. Advanced EEG analysis, such as spatiotemporal decomposition SEDACA have been proposed to provide more objective analysis. The SEDACA (short epoch, dominant activity, cluster analysis) is a type of spatio-temporal decomposition of the EEG signal which allows activity from sources with smaller amplitude to become dominant, first by clustering the points representing epoch dominated by activity from the same source and then by removing the corresponding temporal activity. The SEDACA-derived spectral estimates correlated with neuropsychiatric status and allowed the differentiation of patients with minimal hepatic encephalopathy from the reference population [41]. These types of analysis require specialised software and skills; therefore, they are not routinely used in clinics.

**Table 2:** Characteristics of the most frequently used diagnostic methods for minimal hepatic encephalopathy.

Test	Sensitivity (%)	Specificity (%)	AUROC (%)	Ease of Use	Time Requirement	Gold Standard Used for Comparison
PHES [20,22]	45–57	85–97	-	Simple	10–20 min	EEG
ANT [37]	78.0	63.0	-	Simple	1 min	PHES
Stroop test [32]	94.0	79.0	91.0	Simple	<5 min [42]	SPTs
CFF [43]	61.0	79.0	84.0	Intermediate [42]	5–15 min [42]	This was a meta-analysis
ICT [44]	87.0	77.0	90.2	Intermediate [42]	14 ± 3	SPTs
CRT [39]	93.0	92.0	-	Intermediate	10 min	Missing

PHES consists of five different paper-pencil tests: number connection test A, number connection test B, serial dotting test, digit symbol test, and a line tracing test. Each test is completed in a shorter time interval when administrated alone; however, reports of classification performance of each individual test are limited. In the case of CFF, results of meta-analysis are cited instead of the best diagnostic performance reported in all other instances. SPTs: standard psycho-metric tests.

## 5. Artificial Intelligence

All current methods of psychometric evaluation of HE, be it pen-and-paper or computer-assisted approaches have two major shortcomings. First, the classical analysis of the obtained data is limited to a relatively small number of discrete predictors, and does not use full spectrum of obtainable information, particularly variables difficult to quantify, such as speech abnormalities, fine motoric changes, etc. In other words, classical PHES tests are designed to describe brain function using only a simplified set of variables. Second, they evaluate changes in brain function on a set of artificially created problems. This approach, although valid, carries an inherent bias because of differences in baseline intelligence, brain flexibility and adaptability in certain subpopulations, which is particularly obvious in lower scores of PHES tests in healthy but elderly people. On the other side, an improvement of PHES in repeated testing among healthy controls presents another form of bias, because it represents a learning effect [21]. Various methods of artificial intelligence can overcome both shortcomings, as they can evaluate normal behaviour and can explore patterns in vast amounts of fuzzy data. For example, artificial intelligence could be theoretically used to analyse (1) the morphological and physiological changes in central nervous system (as describe below), (2) electrophysiological changes registered by electroencephalography [45], and (3) to evaluate motoric functions (e.g., handwriting or speech).

In recent years, artificial intelligence and machine learning have penetrated into many areas of medicine and clinical practice. Machine learning algorithms can learn from data, analyse huge number of recordings containing a high number of variables and find the variables that are important for some outcome. Most importantly, machine learning is able to discover complex and non-linear relationships between variables.

The potential of the application of artificial intelligence to clinical practice is underlined by a recently published review article focusing on the convergence of human and artificial intelligence [46] and an update of the widely accepted TRIPOD statement—TRIPOD-AI [47]. Furthermore, two comprehensive review articles focusing on the application of artificial intelligence to gastroenterology and hepatology have already been published [48,49]. However, none of them discusses artificial intelligence application to minimal hepatic encephalopathy diagnosis. Several studies have been recently performed using different branches of artificial intelligence for diagnosing MHE, although, they have been performed only in research settings thus far.

### 5.1. Existing Application of Artificial Intelligence to MHE Diagnosis

Chen et al. performed brain diffusion tension imaging on 36 cirrhotic patients without hepatic encephalopathy and 29 cirrhotic patients with MHE to measure microstructural integrity and water movement across cell membranes in the white matter. MHE was diagnosed with an abnormal score on any of the three following neuropsychological tests: number connection test A, digit symbol test, and block design test. The authors first segmented T1-weighted brain images to white matter, grey matter, and cerebrospinal fluid. After spatial normalization, they used a Bayesian machine-learning technique, graphical model-based multivariate analysis and a Naïve-Bayes classifier to identify regions, which distinguished cirrhotic patients with minimal hepatic encephalopathy from those with no hepatic encephalopathy (NHE). This analysis identified two spatially distributed white matter regions (predominantly located in bilateral frontal lobes, corpus callosum, and parietal lobes) with a significant potential for diagnosing cirrhotic patients with MHE (the accuracy of measuring the water movement across cell membranes was 80%, sensitivity = 75.9%, specificity 83.3%, the accuracy of measuring microstructural integrity was 92.3%, sensitivity = 100%, and specificity = 83.1%) [50].

Furthermore, Chen et al. used magnetic resonance to acquire brain images of 24 cirrhotic patients with MHE and 29 cirrhotic patients with NHE, while MHE was diagnosed as the PHES ≤ 5. The T1-weighted brain images were segmented into grey matter, white matter, and cerebrospinal fluid. After image postprocessing, a support-vector machine classifier was used to classify cirrhotic patients with and without MHE based on grey matter volumetry. The authors found that decreases and increases in grey matter volume in certain regions were associated with MHE (in the original article, particular regions are described in detail). Their existence may reflect brain structural reorganization due to a chronic liver failure. This approach yielded AUROC as high as 0.94 using leave-one-out cross-validation (sensitivity 83.33%, specificity 82.76%) [51].

Intrinsic brain activity is a neural process unrelated to immediate sensory and motor function and resting-state functional magnetic resonance imaging has emerged as a powerful technique for studying it. Neurons with higher spontaneous activity have a higher consumption of energy (oxygen). This changes the oxyhaemoglobin to deoxyhaemoglobin ratio, which can be detected based on different magnetic susceptibility. In another study, the authors investigated whether the local synchrony of intrinsic brain activity could be used to identify cirrhotic patients with minimal hepatic encephalopathy. They evaluated functional MR scans of 19 cirrhotic patients without hepatic encephalopathy and 16 cirrhotic patients with MHE, which again was diagnosed as the PHES ≤ 5. After acquiring and pre-processing resting-state functional magnetic resonance images, a regional homogeneity analysis was performed to assess the local synchrony of intrinsic brain activity. Then, from the regional homogeneity map, the authors selected features with the most significant discriminative potential and used support-vector machines to classify cirrhotic patients according to the presence of MHE. This model’s accuracy, sensitivity, and specificity were 82.9%, 81.3%, and 84.2%, respectively. The most discriminative regions between MHE and NHE are described in detail [52].

Jiao et al. investigated 43 NHE cirrhotic patients and 33 cirrhotic patients with MHE, diagnosed as impaired with two standard deviations beyond normative performance on at least two of four neuropsychiatric tests (trail making test A, trail making test B, digit symbol test, and block design test). They also used resting-state functional magnetic resonance imaging to measure intrinsic brain activity, which they organized in a set of patterns called intrinsic connectivity networks (ICNs) using spatio-temporal dual regression. Then, they applied a Bayesian machine-learning technique and graphical model-based multivariate analysis to identify intrinsic connectivity network regions that characterized group differences (MHE vs. no hepatic encephalopathy). Finally, to avoid algorithm bias, the authors evaluated the discrimination ability of these regions using three different classifiers: support-vector machines, multilayer perceptrons, and C4.5 [53].

Cheng et al. went further and tested whether the time-varying properties of brain connectivity could discriminate between cirrhotic patients with and without MHE. They hypothesized that dynamic characteristics of functional connectivity (the overall strength, stability, and variation of the nodal degree fluctuation over time) could further improve the performance of magnetic resonance in discriminating cirrhotic patients with NHE from those with MHE. The authors acquired functional magnetic resonance images of 32 patients without hepatic encephalopathy and 30 patients with MHE diagnosed by neuropsychiatric testing (number connection test A and digit symbol test, both adjusted for age and education). After feature selection, the authors used support-vector machines to find that the overall strength of the nodal degree fluctuation with time had the best discrimination accuracy on the leave-one-out cross-validation (72.5%), which was 10.5% higher compared to static features [54].

Similarly, Zhang et al. studied pre-processed resting-state functional magnetic resonance brain images of 32 cirrhotic patients with NHE and 30 cirrhotic patients diagnosed with MHE by neuropsychiatric testing (number connection test A and digit symbol test). The authors created 14 brain functional networks [55] and then obtained the Pearson’s correlation whole-brain functional connectivity matrix. They also tracked dynamic changes in the brain over a short period and created a dynamic functional connectivity matrix (dynamic graph) and correlated dynamic network properties with neuropsychological scores. The authors conclude that node disjointness (the percentage of times a node changes its network affiliation independently) in higher cognitive networks as predictor and support-vector machines algorithm as classifier between no hepatic encephalopathy and MHE yield an accuracy of 88.71% [55].

Most interestingly, Dickerson et al. used natural language processing (a branch of artificial intelligence used by Amazon’s Alexa, Apple’s Siri, and Google Assistant), and a paired Wilcoxon signed-rank test to find that patients with MELD ≥ 30 (model for end-stage liver disease score) had decreased word length and fewer 6+ letters words before liver transplant compared to after liver transplant. Similarly, the sentence length decreased following liver transplant [56].

Most of the diagnostic methods mentioned above employ brain magnetic resonance imaging to diagnose MHE by evaluating microstructural integrity and water movement across cell membranes in the white matter and by calculating grey matter volume. They are also able to indirectly evaluate the immediate intrinsic brain activity and how it changes with time. Machine learning classifiers are able to discriminate between cirrhotic patients with NHE and MHE using that information as input data.

### 5.2. Outlook for the Further Application of Artificial Intelligence to MHE Diagnosis

All methods mentioned above, except the latter, have been developed using magnetic resonance imaging data. However, it would be difficult to transform such diagnostic algorithms into clinical practice because of their economic burden. Therefore, there still is a need for a real-time and cost-effective means to identify MHE in cirrhotic patients that would not be time-consuming. One of the aspects that can be analysed through artificial intelligence is discreet changes in motoric functions. We hypothesize that, similarly to asterixis in OHE (or Parkinson’s disease, in fact), minimal motoric changes that are not apparent to the naked eye are already present in MHE. For example, one study found that ataxia and tremors are early markers for cerebral dysfunction in HE patients before neuropsychiatric alterations become detectable [57].

**Figure 1:**
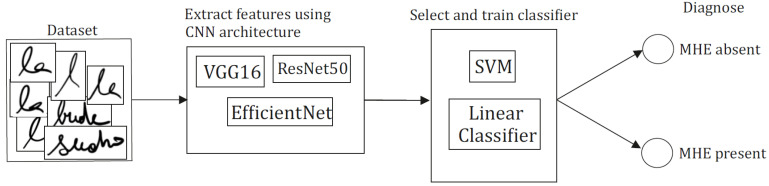
Convolutional neural networks (CNNs) are known for unprecedented performance in various tasks, including computer vision. Deep learning can directly select important features from training data, and it lowers the effort of tedious preprocessing and manual feature selection. Countless convolutional neural network architectures can be used, but they often share similar common concepts as convolutional, pooling, and dense layers. ResNet50 [60], EfficientNet [61], and VGG16 [62] are all considered the state-of-art. Representations can be reused either in linear classification head (group classification based on the value of a linear combination of the characteristics [63]) or traditional machine learning algorithms such as SVM (support-vector machine algorithm [64]), logistic regression, and many others.

As mentioned above, artificial intelligence can explore such changes in multidimensional data obtained either by the recording of handwriting or speech [56,58]. The first step of the proposed algorithm would be extracting features characterizing the underlying patterns of the speech or handwriting signals using signal processing algorithms. The next step would be selecting a subset of these features comprising relevant and minimally overlapping information regarding MHE. Finally, mapping the feature subset to the results of the already established test would take place in a standard supervised learning setup. The proposed workflow is depicted in Figure 1. CNNs are a class of deep neural networks commonly used in artificial intelligence analysis of visual or audio data [59].

Here, we are motivated also by approaches established in diagnosing and monitoring treatment of neurological disorders, such as Parkinson’s disease (PD) and Alzheimer’s disease (AD). Speech and handwriting data are probably the most accessible data for evaluating the cognitive state of the patient. Moreover, speech and handwriting data can be acquired at low cost and without any distress to the patient. Speech has proven to be a suitable modality for diagnosing Alzheimer’s disease [65,66] and Parkinson’s disease [67,68]. In the case of AD, the accuracy of the diagnosis depends on the type of speech features used and computational algorithm employed. Most studies are based on simple vocal and prosodic speech features report accuracy varying from 70 to 90% [69]. Approaches that utilize more complex speech characteristics achieves comparably higher accuracy, with one of the most promising being a classification accuracy around 94% with sensitivity and specificity accuracies of 97% and 91%, respectively [70]. Similarly to AD, PD diagnosis based on voice recordings has been utilized with notable success. The combination of highly sophisticated speech features such as detrended fluctuation analysis, glottal-to-noise excitation and vocal-fold excitation ratio and feature selection methods with a support-vector machines classifier yielded an accuracy of 99% in discriminating PD and healthy control subjects [71]. Other studies have linked the deterioration of parkinsonian speech to PD symptom severity [72,73].

In comparison to speech, handwriting requires less pre-processing and offers similar diagnostic opportunities that proved to be an effective diagnostic modality [74,75,76]. Basically, there are two main approaches to the processing of handwriting: online handwriting, which utilizes graphical tablets or specialized pens to capture pen tip movement and time stamps, and offline handwriting, relying purely on handwriting represented as an image. By using the online handwriting approach, characteristics of handwriting such as kinematics, pressure and spatial-temporal features can be computed [74]. Kinematic features of handwriting were used to distinguish between AD patients, healthy controls and patients with mild cognitive impairment (MCI), achieving almost 100% specificity and 90% sensitivity in the classification of healthy control vs. an AD+MCI group [77]. Regarding PD, in addition to slowness of movement, the reduction of writing size (micrography) is a frequent symptom, which also makes it more suitable for offline handwriting processing. The highest achieved results on the classification of PD subjects from healthy controls were achieved by a support-vector machine classifier for online handwriting, which provided 98% accuracy [78].

We believe that such a trained convoluted neural network would require a simple smartphone recording only. This process would be fast and fully automatic, sparing a vast amount of physician’s time compared to a PHES test battery that takes about 20 min to complete. Such a fast and fully automatic method could finally be used in the point-of-care setting and, after extensive external validation, possibly even in the self-monitoring of cirrhotic patients, for instance, before driving a car.

## 6. Conclusions

Minimal hepatic encephalopathy is a complication of liver failure (or portosystemic shunting) associated with decreased health-related quality of life, worse prognosis, and decreased survival in patients with liver cirrhosis. Lifestyle changes and pharmacological treatment can reverse minimal hepatic encephalopathy. Therefore, it is essential to regularly screen patients with liver cirrhosis for its presence. Currently, time-consuming paper-and-pencil tests or the Stroop test are rarely being used in clinical practice. Different artificial intelligence algorithms were employed in research settings to diagnose minimal hepatic encephalopathy from brain magnetic resonance images. However, the cost-effective and fully automatic analysis of common behavioral patterns such as handwriting or speech could pave the way to increasing the rate of minimal hepatic encephalopathy screening in clinical practice.

## Figures and Tables

**Table 1:** Clinical presentation of hepatic encephalopathy.

ISHEN	WHC	Description	Suggested Operative Criteria
Unimpaired		No Encephalopathy at All, No History of HE	Tested and Proved to be Normal
Covert	Minimal	Psychometric or neuropsychological alterations of tests exploring psychomotor speed/executive functions or neurophysiological alterations without clinical evidence of mental change.	Abnormal results of established psychometric or neuropsychological tests without clinical manifestations
	Grade I	Trivial lack of awareness Euphoria or anxiety Shortened attention span Impairment of addition or subtraction Altered sleep rhythm	Despite oriented in time and space (see below), the patient appears to have some cognitive/behavioural decay with respect to his/her standard on clinical examination, or to the caregivers
Overt	Grade II	Lethargy or apathy Disorientation for time Obvious personality change Inappropriate behaviour Dyspraxia Asterixis	Disoriented for time (at least three of the followings are wrong: day of the month, day of the week, month, season or year) ± the other mentioned symptoms
	Grade III	Somnolence to semi-stupor Responsive to stimuli Confused Gross disorientation Bizarre behaviour	Disoriented also for space (at least three of the following wrongly reported: country, state [or region], city or place) ± the other mentioned symptoms
	Grade IV	Coma	Does not respond even to pain stimuli

Note: Adapted from [1].
